# Classifying Firearm Injury Intent in Electronic Hospital Records Using Natural Language Processing

**DOI:** 10.1001/jamanetworkopen.2023.5870

**Published:** 2023-04-06

**Authors:** Erin MacPhaul, Li Zhou, Stephen J. Mooney, Deborah Azrael, Andrew Bowen, Ali Rowhani-Rahbar, Ravali Yenduri, Catherine Barber, Eric Goralnick, Matthew Miller

**Affiliations:** 1 Department of Emergency Medicine, Brigham and Women's Hospital, Harvard Medical School, Boston, Massachusetts; 2 Firearm Injury & Policy Research Program, University of Washington, Seattle; 3 Department of Epidemiology, School of Public Health, University of Washington, Seattle; 4 Harvard Injury Control Research Center, Harvard T. H. Chan School of Public Health, Boston, Massachusetts; 5 Center for Surgery and Public Health, Brigham and Women’s Hospital, Boston, Massachusetts; 6 Department of Health Sciences, Bouve College of Health Sciences, Northeastern University, Boston, Massachusetts

## Abstract

**Importance:**

*International Classification of Diseases*–coded hospital discharge data do not accurately reflect whether firearm injuries were caused by assault, unintentional injury, self-harm, legal intervention, or were of undetermined intent. Applying natural language processing (NLP) and machine learning (ML) techniques to electronic health record (EHR) narrative text could be associated with improved accuracy of firearm injury intent data.

**Objective:**

To assess the accuracy with which an ML model identified firearm injury intent.

**Design, Setting, and Participants:**

A cross-sectional retrospective EHR review was conducted at 3 level I trauma centers, 2 from health care institutions in Boston, Massachusetts, and 1 from Seattle, Washington, between January 1, 2000, and December 31, 2019; data analysis was performed from January 18, 2021, to August 22, 2022. A total of 1915 incident cases of firearm injury in patients presenting to emergency departments at the model development institution and 769 from the external validation institution with a firearm injury code assigned according to *International Classification of Diseases*, *Ninth Revision, Clinical Modification* (*ICD-9-CM*) or *International Statistical Classification of Diseases and Related Health Problems, 10th Revision, Clinical Modification* (*ICD-10-CM*), in discharge data were included.

**Exposures:**

Classification of firearm injury intent.

**Main Outcomes and Measures:**

Intent classification accuracy by the NLP model was compared with *ICD* codes assigned by medical record coders in discharge data. The NLP model extracted intent-relevant features from narrative text that were then used by a gradient-boosting classifier to determine the intent of each firearm injury. Classification accuracy was evaluated against intent assigned by the research team. The model was further validated using an external data set.

**Results:**

The NLP model was evaluated in 381 patients presenting with firearm injury at the model development site (mean [SD] age, 39.2 [13.0] years; 348 [91.3%] men) and 304 patients at the external development site (mean [SD] age, 31.8 [14.8] years; 263 [86.5%] men). The model proved more accurate than medical record coders in assigning intent to firearm injuries at the model development site (accident F-score, 0.78 vs 0.40; assault F-score, 0.90 vs 0.78). The model maintained this improvement on an external validation set from a second institution (accident F-score, 0.64 vs 0.58; assault F-score, 0.88 vs 0.81). While the model showed some degradation between institutions, retraining the model using data from the second institution further improved performance on that site’s records (accident F-score, 0.75; assault F-score, 0.92).

**Conclusions and Relevance:**

The findings of this study suggest that NLP ML can be used to improve the accuracy of firearm injury intent classification compared with *ICD*-coded discharge data, particularly for cases of accident and assault intents (the most prevalent and commonly misclassified intent types). Future research could refine this model using larger and more diverse data sets.

## Key Points

**Question:**

Is use of natural language processing and machine learning techniques associated with improved accuracy in firearm injury intent coding in hospital discharge data?

**Findings:**

In this cross-sectional study using a total of 2684 cases of firearm injury, a natural language processing model was developed using free-text notes in the electronic health record to assign intent to firearm injury incidents in patients presenting to emergency departments. The model proved more accurate in identifying the intent of firearm injuries than did the *International Classification of Diseases* codes assigned by medical records coders at the model development site and maintained this advantage on an external validation set of 304 cases from a second institution.

**Meaning:**

The findings of this study suggest that natural language processing and machine learning techniques as applied to free-text electronic health record data are associated with improved accuracy of intent coding commonly relied on for firearm injury surveillance and research.

## Teaser

This cross-sectional study examines the development of a machine learning model using natural language processing to classify the intent of firearm-related injury.

## Introduction

An expert panel recently characterized the absence of reliable data about the underlying intent of firearm injuries presenting to US emergency departments as a notable gap in the US firearms surveillance infrastructure.^1^ The main reason for this deficiency is that while hospital discharge data capture a census of such injuries presenting to US emergency departments, intent coding, which specifies whether a gunshot injury resulted from assault, accident, self-harm, legal intervention, or is of unknown intent, is grossly inaccurate.^2,3,4,5^As a result, peer-reviewed studies that have used *International Classification of Diseases* (*ICD*)-coded discharge data to assess firearm injuries by intent, including over a dozen published in high-impact journals over the past 6 years, inaccurately characterize the burden of and trends in intent-specific firearm injuries.^6,7,8,9,10,11,12,13,14,15,16^

Natural language processing (NLP) and machine learning (ML) have been used to efficiently identify information contained in free-text narratives, including clinical notes and reports in electronic health records (EHRs).^17^ Because these approaches have the potential to accurately assign firearm injury intent and thus result in more accurate surveillance of these injuries, we developed a hybrid model using NLP and ML to classify intent from free-text narratives in the EHR. We then evaluated the accuracy of the model at the model development site (MDS) and at another level I trauma center, the external validation site (EVS), using researcher-adjudicated intent as the highest standard.

## Methods

### Clinical Setting, Patient Population, and Data Sources

This study used EHR data (eg, visit notes, progress notes, and discharge summaries) of all patients with firearm injuries who were admitted to the emergency departments of 3 level I trauma centers at 2 large integrated health care systems, one in Boston, Massachusetts, between January 1, 2000, and December 31, 2019 (the MDS), and the other in Seattle, Washington, between March 20, 2016, and December 31, 2018 (the EVS). The study was approved by the research ethics board at the Brigham and Women’s Hospital and by the human subjects division at the University of Washington. The need for informed consent was waived owing to secondary use of EHR data. This study follows the Strengthening the Reporting of Observational Studies in Epidemiology (STROBE) reporting guideline for observational studies.

Firearm injuries of any intent were identified in the centralized data repository from the MDS hospital system by requesting patient encounters assigned a firearm injury code in any diagnosis or external cause of injury field according to the *International Classification of Diseases*, *Ninth Revision, Clinical Modification* and the *International Statistical Classification of Diseases and Related Health Problems, 10th Revision, Clinical Modification* (hereafter, *ICD* indicates both) (eAppendix 2 in Supplement 1 provides a comprehensive list of *ICD* codes used).

### Development of Data Sets

For all patient encounters assigned a firearm-related *ICD* code, members of the research team manually reviewed the EHR to ensure the case was an initial presentation seen in the emergency department, filtering out cases that were follow-up visits for an earlier injury, cases in which the patient’s injury did not result from firearm discharge (eg, pistol whipping), and cases miscoded as a firearm injury (eg, airgun or BB gun injury). If a person was shot in more than 1 incident during the project period, each incident was considered a separate case. We assigned injury intent based on a detailed coding manual developed for the overarching project of which this study is a component; the codebook is available in Supplement 1.^7^ Administrative data on self-reported race and ethnicity (Black, Hispanic, White, other racial or ethnic minority group [including American Indian or Alaska Native, Asian, Middle Eastern or North African, Native Hawaiian or other Pacific Islander, and multiple races or ethnicities], or unknown or missing) were used to illustrate how the burden of intent-specific firearm injury differed across the model development and external evaluation sites. A total of 1915 cases were included of 2313 potential cases examined at the MDS and 769 cases of 964 potential cases at the EVS. This manual adjudication process occurred from January 18, 2021, through January 3, 2022. A 10% sample was coded by more than 1 team member early in the coding process; coding disagreement was low (5%), resolved by discussion, and the codebook was iteratively updated to reduce subjectivity.

To develop the NLP algorithm, we first split the MDS data into training, tuning, and test sets with a 60%/20%/20% split. To ensure that prior knowledge of the test set cases did not contaminate model development, we split the cases into these sets before manually coding intent. Team members involved in the development of the classification models did not review test cases.

### Model Development

Our NLP model classifies firearm injury intent in 3 steps. First, sentences containing key terms are extracted from the narrative health records. Second, these extracted sentences are processed by a rule system to identify which rules apply to the case. These rules aim to capture the intent-relevant information reflected by our expert domain knowledge and detailed coding manual. Third, these rules are used as binary features and processed by an ML classifier to assign an intent label to each case. The trained model can be saved and used on new data without needing to retrain the model and can also be easily retrained, which allows flexibility when encountering new data. We implemented the model using Python, version 3.10.5 (Python Software Foundation).

#### Lexicon Creation and Key Term Extraction

We first created a specialized lexicon for identifying sentences with potential injury intent information. The lexicon consists of terms defined by a word or phrase and a category. The initial set of terms was derived from the team’s experience in firearm injury surveillance and reviewing cases in the training data sets. We expanded the lexicon with a hand-picked selection of relevant terms between 1 and 5 words long identified through term frequency-inverse document frequency scores, which represent the ratio of frequency of a term in the cases of each intent to how common the term was in the overall data set. We added new lexicon terms as needed while refining the rules and examining classification errors on the tuning set, including some added to prevent the model from confusing similar terms. These terms, shown under the confusion terms category in eTable 1 in Supplement 1, let the model distinguish between an informative term and an uninformative term that happens to have overlapping words. For example, by including *police report* as a confusion term, the model knows not to consider instances of the term *police report* when looking for the term *police* to determine whether police officers were involved in the shooting, as police reports are often cited for all types of intent.

eTable 1 in Supplement 1 lists the term categories and examples of the terms included by category. Terms can also have attribute tags, which are extra pieces of information about the term for the rules to use (eg, whether the incident location term is *inside* or *outside*). Tags can be seen in the full lexicon (see supplementary materials).

With this lexicon, our NLP tool MTERMS searches the EHR for the lexicon terms and extracts the sections of the EHR containing these key terms.^18,19^ MTERMS additionally extracts key contextual information about the use of each of the terms in the lexicon, such as the sentence and clinical note section that contained the term and whether specified modifying terms appear, such as negations or indicators that the term is a historical condition or a family member’s condition. For each sentence with a term from the shooting verbs category, such as *shot* and *fired*, a dependency parse using the SPACY NLP library is created and checked for specific grammatical relations to extract the subject of the shooting verbs as the shooter, the object of the verb as the person shot, and some adverbial and prepositional phrases as potential circumstances of the shooting.

#### Rule Set Development

Next, we designed a set of rules to classify intent. Each rule processes the terms, term categories and attribute tags, contextual information, and dependency parse extracts to determine whether the rule applies to the case. Some rules, such as the explicit intent rules, check for the presence of a specific term or term category. Other rules, such as the assault shooter rule, require more complex logic including attribute tags and the dependency parse extracts. eTable 2 in Supplement 1 provides a comprehensive list of rules in the rule system. We developed the rule system using the MDS training set.

Unknown intent provides a unique challenge, as unknown cases are defined by their lack of information, which cannot be extracted from text with key terms and rules. Some unknown cases have explicit attestations of a lack of information, eg, the intent of the shooting is unknown, but most simply lack any intent information. To deal with this, some rules are based on the amount of information present rather than the specific information that was found, such as the high info note and low info case rules, which compare the number of unique term and context pairs extracted by MTERMS against a threshold value manually tuned to optimize unknown intent classification in MDS tuning data. Other rules, such as the no explicit intent and conflicting intent rules, check how many explicit intent rules returned true.

#### ML Classifiers

The results from the rule system are used as a set of binary features to train an ML model to determine a final intent label for the case. These models assign a probability for each intent based on the feature patterns of the training data; the highest probability intent is assigned to the case. We used the Scikit-Learn Gradient Boosting Classifier model after it returned the highest unweighted average F-score results during testing compared with random forest, maximum entropy, support vector machines, ada boost, and k-nearest neighbor algorithms.^20^ We used the MDS tuning set to select the ML model and tune the model parameters.

The performance of the different classification algorithms is summarized in eTable 3 in Supplement 1. The Scikit-Learn Gradient Boosting Classifier achieved the highest performance on the tune set, with an unweighted average F-score of 79.1%, area under the receiver operating characteristic curve (AUROC) of 0.921, and area under the precision-recall curve of 0.772. This was achieved with parameters set to a maximum depth of 1, a learning rate of 0.1, a tree count of 300, and otherwise default values.

### Evaluation

We evaluated the performance of the classification models on an untouched MDS test set with standard metrics including specificity ([true negatives] / [true-negatives + false-positives]); recall, aka sensitivity: ([true-positives] / [true-positives + false-negatives]; precision, aka positive predictive value: ([true-positives] / [true-positives + false-positives]); F-score = 2 × (precision × recall) / (precision + recall); AUROC; and, because AUROC can mask large differences in the rate of false-positives on unbalanced data sets, we also computed area under the precision-recall curve.

### External Validation

We evaluated the performance of the classification model developed at the MDS externally on EHR data from gunshot injury incidents that met our case definition at the EVS, using the same metrics applied for internal evaluation of the MDS test data. We divided data at the EVS into training and test data sets with a 60%/40% split. We first tested performance of the best classification model developed with MDS data on the EVS data. We then recalibrated the model using EVS training data and evaluated the performance of the recalibrated EVS model using the MDS test data as an external validation set to assess loss of accuracy due to between-site differences.

## Results

The final training, tuning, and test data sets at the MDS and the EVS are described in Table 1. Across all sites, most patients were male and had a mean age between 30 and 40 years. Of MDS patients included in the test set, mean (SD) age was 39.2 (13.0) years, 348 (91.3%) were men, and 33 (8.7%) were women. Of EVS patients in the test set, mean (SD) age was 31.8 (14.8) years, 263 (86.5%) were men, and 41 (13.5%) were women. The MDS cases had relatively more researcher-adjudicated assaults and unknown intents, while a larger proportion of EVS cases were adjudicated to be self-harm or accidental gunshot injuries. A larger percentage of cases were labeled accidents and a smaller percentage were labeled assaults by medical record coders at both sites compared with the researcher-adjudicated intents.

**Table 1.** Characteristics of Firearm Injury Cases at MDS and EVS

Characteristic	No. (%)						
	MDS				EVS		
	Training set	Tuning set	Test set	Total	Training set	Test set	Total
Case count	1146	388	381	1915	465	304	769
Age, mean (SD), y	38.0 (14.0)	37.5 (13.9)	39.2 (13.0)	38.1 (13.8)	31.5 (14.0)	31.8 (14.8)	31.6 (14.3)
Sex							
Female	74 (6.5)	33 (8.5)	33 (8.7)	140 (7.3)	58 (12.5)	41 (13.5)	99 (12.9)
Male	1072 (93.5)	355 (91.5)	348 (91.3)	1775 (92.7)	406 (87.3)	263 (86.5)	669 (87.0)
Unknown	0	0	0	0	1 (0.2)	0	1 (0.1)
Race							
White	291 (25.4)	94 (24.2)	101 (26.5)	486 (25.4)	227 (48.8)	146 (48.0)	373 (48.5)
Black	414 (36.1)	131 (33.8)	139 (36.5)	684 (35.7)	159 (34.2)	94 (30.9)	253 (32.9)
Asian	48 (4.2)	15 (3.9)	11 (2.9)	74 (3.9)	22 (4.7)	19 (6.3)	41 (5.3)
Other^a^	274 (23.9)	100 (25.8)	86 (22.6)	460 (24.0)	48 (10.3)	36 (11.8)	84 (10.9)
Unknown	119 (10.4)	48 (12.4)	44 (11.5)	211 (11.0)	9 (1.9)	9 (3.0)	18 (2.3)
Ethnicity							
Hispanic	294 (25.7)	118 (30.4)	105 (27.6)	517 (27.0)	70 (15.1)	64 (21.1)	134 (17.4)
Non-Hispanic	375 (32.7)	125 (32.2)	129 (33.9)	629 (32.8)	395 (85.0)	240 (78.9)	635 (82.6)
Unknown	477 (41.6)	145 (37.4)	147 (38.6)	769 (40.2)	0	0	0
Researcher-adjudicated intent							
Accident	88 (7.7)	28 (7.2)	27 (7.1)	143 (7.5)	67 (14.4)	47 (15.5)	114 (14.8)
Assault	799 (69.7)	272 (70.1)	285 (74.8)	1356 (70.8)	311 (66.9)	200 (65.8)	511 (66.4)
Legal intervention	19 (1.7)	8 (2.1)	8 (2.1)	35 (1.8)	17 (3.7)	11 (3.6)	28 (3.6)
Self-harm	66 (5.8)	24 (6.2)	14 (3.7)	104 (5.4)	53 (11.4)	35 (11.5)	88 (11.4)
Unknown^b^	174 (15.2)	56 (14.4)	47 (12.3)	277 (14.5)	17 (3.7)	11 (3.6)	28 (3.6)
*ICD*-coded intent in discharge data^c^							
Accident	254 (24.4)	78 (21.9)	77 (22.3)	409 (23.5)	165 (35.5)	96 (31.6)	261 (33.9)
Assault	584 (56.0)	200 (56.2)	207 (60.0)	991 (56.9)	225 (48.4)	146 (48.0)	371 (48.2)
Legal intervention	10 (1.0)	4 (1.1)	6 (1.7)	20 (1.1)	15 (3.2)	8 (2.6)	23 (3.0)
Self-harm	55 (5.3)	16 (4.5)	13 (3.8)	84 (4.8)	45 (9.7)	40 (13.2)	85 (11.1)
Unknown^b^	139 (13.3)	58 (16.3)	42 (12.2)	239 (13.7)	15 (3.2)	14 (4.6)	29 (3.8)

Abbreviations: EHR, electronic health record; EVS, external validation site; *ICD*, *International Classification of Diseases*; MDS, model development site.

^a^ Includes patients whose race and ethnicity was recorded in the EHR as American Indian or Alaska Native, 2 or more, and other.

^b^ Includes credibly conflicting and undetermined intents. Codes were assigned according to *International Classification of Diseases*, *Ninth Revision, Clinical Modification*, or *International Statistical Classification of Diseases and Related Health Problems, 10th Revision, Clinical Modification*.

^c^ A total of 91% of cases at MDS and all cases at EVS had *ICD*-intent codes in discharge data assigned by medical records coders for hospital billing.

### Model Performance at MDS

Table 2 presents the model-assigned intent compared with the intent associated with the *ICD* code assigned by medical record coders in discharge data for each site. At the MDS, the NLP model outperformed the *ICD*-assigned intent for accidents, assaults, and unknowns, while maintaining the same performance for self-harm cases. Moreover, for the most commonly misclassified classes of firearm injury intents (assaults and accidents), the NLP model was far less likely to result in false-positive accidental injury labels than the *ICD* intent (precision, 0.73 vs 0.26; F-score, 0.78 vs 0.40) and meaningfully more likely to label an assault correctly (recall, 0.91 vs 0.70; F-score, 0.90 vs 0.78). Because our data set had a very low number of legal intervention cases, the model was not able to meaningfully learn to identify these cases. Excluding legal intervention cases, the model maintained its performance between the tune cases and the previously unseen test set, suggesting rules were not overtuned toward the tune set.

**Table 2.** Intent-Level Performance for Best Multilevel NLP Model Developed at the MDS vs *ICD*-Coded Intent Assigned by EHR Coders in Discharge Data at the MDS and at EVS^a^^,^^b^

Variable	Accident		Assault		Legal intervention		Self-harm		Unknown	
	*ICD*	NLP	*ICD*	NLP	*ICD*	NLP	*ICD*	NLP	*ICD*	NLP
**MDS test set**										
Specificity	0.82	0.98	0.72	0.63	0.99	1.00	0.99	0.98	0.88	0.94
Precision	0.26	0.73	0.89	0.89	0.67	0.50	0.69	0.59	0.14	0.45
Recall	0.87	0.83	0.70	0.91	0.67	0.17	0.90	1.00	0.14	0.33
F-score	0.40	0.78	0.78	0.90	0.67	0.25	0.78	0.74	0.14	0.38
AUROC	NA	0.98	NA	0.89	NA	0.96	NA	0.99	NA	0.84
AUPRC	NA	0.76	NA	0.96	NA	0.45	NA	0.83	NA	0.43
**EVS test set**										
Specificity	0.78	0.93	0.96	0.70	1.00	0.99	0.92	0.97	0.95	0.99
Precision	0.43	0.62	0.97	0.87	0.91	0.60	0.87	0.79	0	0.33
Recall	0.87	0.66	0.70	0.89	0.59	0.27	0.98	0.94	0	0.09
F-score	0.58	0.64	0.81	0.88	0.71	0.38	0.92	0.86	0	0.14
AUROC	NA	0.89	NA	0.89	NA	0.95	NA	0.99	NA	0.73
AUPRC	NA	0.72	NA	0.93	NA	0.43	NA	0.89	NA	0.15
AUPRC	NA	0.79	NA	0.95	NA	0.57	NA	0.84	NA	0.09

Abbreviations: AUPRC, area under the precision-recall curve; AUROC, area under the receiver operating characteristic curve; EVS, external validation site; *ICD*, *International Classification of Diseases*; MDS, model development site; NLP, natural language processing.

^a^ Within the MDS data set, 9% of cases were manually validated as meeting our case criteria but lacked a hospital-assigned discharge code. These 9% of cases were excluded from analyses presented in Table 2, allowing NLP model performance (against our highest standard, researcher-adjudicated intent) to be compared with the performance of *ICD*-coded discharge data (against researcher-adjudicated intent) using the same cases.

^b^ Codes were assigned according to *International Classification of Diseases*, *Ninth Revision, Clinical Modification*, or *International Statistical Classification of Diseases and Related Health Problems, 10th Revision, Clinical Modification*.

### Model Performance at EVS

Table 2 presents data showing that our model classified external validation cases without new training data and still outperformed the *ICD*-coded intent in discharge data. While the results degraded slightly between institutions, our model maintained its advantage against the EVS *ICD* codes at more precisely labeling accidental gunshot injuries (precision 0.62 vs 0.43; F-score, 0.64 vs 0.58) while still outperforming the *ICD* codes at identifying assaults (recall 0.89 vs 0.70; F-score, 0.88 vs 0.81).

As Table 3 indicates, the degradation in performance for assaults and accidents by the MDS model on the EVS data can be ameliorated by retraining the model with local data. The MDS-trained model and the EVS-trained model performed very similarly for unintentional injury (F score, 0.76 vs 0.75), assaults (F score, 0.89 vs 0.92), and on average (F score, 0.58 vs 0.60) when evaluated against their source institution’s test set. This, and the stability of the self-harm results for each institution regardless of the model (F score, 0.73 vs 0.67 on the MDS data set and 0.86 vs 0.82 on the EVS data set), suggests our rule system is not overly designed toward the characteristics of the MDS data and can still capture the important features of the external data.

**Table 3.** Intent Level Results for Best Models Applied to Test Data From the MDS and EVS After Initial Training at MDS and After Recalibration at EVS

Variable	Accident		Assault		Legal intervention		Self-harm		Unknown		Average	
	MDS	EVS	MDS	EVS	MDS	EVS	MDS	EVS	MDS	EVS	MDS	EVS
**Model trained on MDS data**												
Specificity	0.98	0.93	0.59	0.70	0.99	0.99	0.98	0.96	0.95	0.99	0.50	0.51
Precision	0.75	0.62	0.87	0.87	0.33	0.60	0.63	0.79	0.45	0.33	0.61	0.64
Recall	0.78	0.66	0.92	0.89	0.13	0.27	0.86	0.94	0.30	0.09	0.59	0.57
F-score	0.76	0.64	0.89	0.88	0.18	0.38	0.73	0.86	0.36	0.14	0.58	0.58
AUROC	0.97	0.89	0.89	0.89	0.96	0.95	0.99	0.99	0.84	0.73	0.93	0.89
AUPRC	0.77	0.72	0.96	0.93	0.45	0.43	0.81	0.89	0.42	0.15	0.68	0.62
**Model trained on EVS data**												
Specificity	0.99	0.98	0.42	0.71	0.98	0.99	0.99	0.98	0.99	1.00	0.48	0.58
Precision	0.78	0.84	0.83	0.87	0.30	0.71	0.69	0.85	0.25	0	0.57	0.65
Recall	0.63	0.68	0.97	0.96	0.38	0.45	0.64	0.80	0.02	0	0.53	0.57
F-score	0.69	0.75	0.89	0.92	0.33	0.56	0.67	0.82	0.04	0	0.53	0.60
AUROC	0.93	0.91	0.88	0.93	0.97	0.97	0.97	0.98	0.72	0.71	0.89	0.90
AUPRC	0.67	0.79	0.95	0.95	0.35	0.57	0.72	0.84	0.34	0.09	0.61	0.65

Abbreviations: AUPRC, area under the precision-recall curve; AUROC, area under the receiver operating characteristic curve; EVS, external validation site; MDS, model development site.

### Error Analysis

The main sources of error for the MDS model on the MDS test set are the unknown labels (Table 4). The unknowns present 2 sources of error for our model: true unknowns falsely labeled as assault and true assaults falsely labeled as unknown. The main feature behind both errors is the high info note rule, which relies on a high info threshold manually tuned to capture the MDS unknown cases, which are more common in the earlier years of our data. However, this rule did not account for the kind of changes that occurred at the MDS over time, such as the development of EHR systems, which increased the amount of documentation as more data could be stored electronically but did not affect the amount of unique relevant information. This resulted in cases with equal amounts of unique information but different documentation styles being treated differently by the high info note rule. Results from the EVS, which contained data from recent years only and used the same EHR system throughout the course of our study, corroborated this observation: the model ruled that every test set case at EVS had a high information note. eTable 4 in Supplement 1 reports how this discrepancy affected the ML training process, as the MDS model highly values the high info rule while the EVS model places little value on it, suggesting that our information density-based rules were particularly susceptible to accuracy drift due to institutional changes in the record-keeping process—either over time or across institutions.

**Table 4.** Confusion Matrices and Intent Distributions for the NLP Model vs MDS and EVS Test Data

MDS-trained NLP predictions	No.					No. (%)^a^	
	Accident	Assault	Legal intervention	Self-harm	Unknown	Researcher-adjudicated intent distribution	NLP-labeled intent distribution
**MDS researcher-adjudicated intent set**							
Accident	21	4	0	0	3	27 (7.1)	28 (7.3)
Assault	3	261	5	2	29	285 (74.8)	300 (78.7)
Legal intervention	1	1	1	0	0	8 (2.1)	3 (0.8)
Self-harm	1	3	2	12	1	14 (3.7)	19 (5.0)
Unknown	1	16	0	0	14	47 (12.3)	31 (8.1)
**EVS researcher-adjudicated intent set**							
Accident	31	14	0	2	3	47 (15.5)	50 (16.4)
Assault	12	178	8	0	6	200 (65.8)	204 (67.1)
Legal intervention	0	2	3	0	0	11 (3.6)	5 (1.6)
Self-harm	3	5	0	33	1	35 (11.5)	42 (13.8)
Unknown	1	1	0	0	1	11 (3.6)	3 (1.0)

Abbreviations: EVS, external validation site; MDS, model development site; NLP, natural language processing.

^a^ MDS researcher-adjudicated intent set, n = 381; EVS researcher-adjudicated intent set, n = 304.

Small sample sizes also affected our model’s ability to learn to identify legal intervention and, to a lesser extent, self-harm. These intents frequently share features with assault cases, and the latter with accidents as well. As a result, the model was unable to learn to distinguish these intents as well as were human coders.

## Discussion

We implemented an NLP model to identify firearm injury intent in narrative text using ML. Our results suggest that an NLP model based on an expert-constructed rule set can classify firearm intent more accurately than extracting *ICD* codes from discharge data.

Compared with reusing *ICD* codes logged by an EHR coder, our approach identified accidents more precisely and improves recall for assaults, even on external data. Our model developed on MDS data performed well at the EVS, suggesting that it, or more refined iterations that might be produced by training on larger data sets, could be used to assist research on other external data sets without requiring retraining on local data. Prior work with individual-level data used in the present study found that trauma registrars and research team members assigned intent similarly, suggesting that a good next step for additional model training would be to use trauma registries as a source of accurately coded intent data more diverse and much larger than those used in the present study.^7^ Our model, especially if retrained on these larger data sets, might be of practical value for research teams and for hospitals that want to retrospectively recode their firearms data but lack the resources to create manually labeled local training and test sets. If feasible, however, training the model on local data would likely still be valuable as our locally trained model generally outperformed the externally trained model on intents for which the model had sufficient training data. Researchers who lack the expertise to retrain the model on local data could still use our model to target manual review (eg, by limiting review to cases in which the model disagrees with the *ICD* intent), thereby augmenting model performance with efficiently deployed human effort.

### Limitations

This study has limitations. Our work builds on recent NLP work by Parker^21^ that used a similar approach to abstract narrative text in the National Electronic Injury Surveillance System–Firearm Injury Surveillance Study to identify where firearm injury incidents occurred. The success of these approaches shows the potential ML has to improve the accuracy and completeness of data commonly used for firearm injury surveillance and research. The current proof-of-concept study should nevertheless be interpreted with some key limitations in mind.

Although we had access to nearly 3000 cases from 2 large health care systems, we were unable to use the EVS data while developing our lexicon and rules due to institutional data sharing restrictions. We were also unable to evaluate a model trained on data from both the MDS and EVS, which would have allowed us to see whether such a multi-institutional model would perform better than did our model. While our model performed well at our EVS, more data from a diverse range of other institutions are needed to further test the model’s reliability with external data. The high info note issue underscores the need for further refinement as it demonstrated that documentation differences between institutions can substantially affect model performance. We suspect model performance would improve if we used a better measure of meaningful information than the information density measure we used, such as the number of rules that are identified as pertaining to a case rather than the number of terms in unique contexts, which would allow the model to ignore redundant information.

## Conclusions

The findings of this study suggest that an ML approach can be used to improve the accuracy of firearm injury surveillance and assist in public health research. Future research should refine our NLP model using larger and more diverse data sets, such as data from trauma registries.

## Notes

Supplement 1. **eAppendix 1.** CODE BOOK (October 5, 2022) **eAppendix 2.** *ICD* Codes Used to Identify Gunshot Injuries **eTable 1.** NLP Lexicon Categories and Sample Terms **eTable 2.** Rule System Descriptions **eTable 3.** Comparison of NLP Models on Tune Set **eTable 4.** Top (and Bottom) 5 Highest (and Lowest) Weighted Rules

Supplement 2.
Data Sharing Statement

## References

[1] NORC at the University of Chicago. Expert panel on firearms data infrastructure. Accessed February 20, 2023. https://www.norc.org/Research/Projects/Pages/expert-panel-on-firearms-data-infrastructure.aspx

[2] Barber C. Improving the Capacity of Hospital ED Data Systems to Track Nonfatal Firearm Injuries. In: Roman J, Cook P, eds. Improving Data Infrastructure to Reduce Firearms Violence. NORC at the University of Chicago; 2021.

[3] Barber C, Goralnick E, Miller M. The problem with *ICD*-coded firearm injuries. JAMA Intern Med. 2021;181(8):1132-1133. doi:10.1001/jamainternmed.2021.0382 33779677

[4] Barber C, Cook PJ, Parker ST. The emerging infrastructure of US firearms injury data. Prev Med. 2022;165(pt A):107129. 35803350 10.1016/j.ypmed.2022.107129

[5] Miller M, Azrael D, Yenduri R, . Assessment of the accuracy of firearm injury intent coding at 3 US hospitals. JAMA Netw Open. 2022;5(12):e2246429. doi:10.1001/jamanetworkopen.2022.46429 36512356 PMC9856424

[6] Kaufman EJ, Delgado MK. The epidemiology of firearm injuries in the US: the need for comprehensive, real-time, actionable data. JAMA. 2022;328(12):1177-1178. doi:10.1001/jama.2022.16894 36166012

[7] Kaufman EJ, Wiebe DJ, Xiong RA, Morrison CN, Seamon MJ, Delgado MK. Epidemiologic trends in fatal and nonfatal firearm injuries in the US, 2009-2017. JAMA Intern Med. 2021;181(2):237-244. doi:10.1001/jamainternmed.2020.6696 33284327 PMC7851729

[8] Patel SJ, Badolato GM, Parikh K, Iqbal SF, Goyal MK. Sociodemographic factors and outcomes by intent of firearm injury. Pediatrics. 2021;147(4):e2020011957. doi:10.1542/peds.2020-011957 33782104

[9] Peek-Asa C, Butcher B, Cavanaugh JE. Cost of hospitalization for firearm injuries by firearm type, intent, and payer in the United States. Inj Epidemiol. 2017;4(1):20. doi:10.1186/s40621-017-0120-0 28721637 PMC5515719

[10] Schnippel K, Burd-Sharps S, Miller TR, Lawrence BA, Swedler DI. Nonfatal firearm injuries by intent in the United States: 2016-2018 hospital discharge records from the Healthcare Cost and Utilization Project. West J Emerg Med. 2021;22(3):462-470. doi:10.5811/westjem.2021.3.51925 34125015 PMC8203029

[11] Kalesan B, Zuo Y, Xuan Z, . A multi-decade joinpoint analysis of firearm injury severity. Trauma Surg Acute Care Open. 2018;3(1):e000139. doi:10.1136/tsaco-2017-000139 29766128 PMC5887778

[12] Avraham JB, Frangos SG, DiMaggio CJ. The epidemiology of firearm injuries managed in US emergency departments. Inj Epidemiol. 2018;5(1):38. doi:10.1186/s40621-018-0168-5 30318556 PMC6186529

[13] Coupet E Jr, Huang Y, Delgado MK. US emergency department encounters for firearm injuries according to presentation at trauma vs nontrauma centers. JAMA Surg. 2019;154(4):360-362. doi:10.1001/jamasurg.2018.4640 30673067 PMC6484801

[14] Kalesan B, Siracuse JJ, Cook A, Prosperi M, Fagan J, Galea S. Prevalence and hospital charges from firearm injuries treated in US emergency departments from 2006 to 2016. Surgery. 2021;169(5):1188-1198. doi:10.1016/j.surg.2020.11.009 33384161

[15] Cook A, Osler T, Hosmer D, . Gunshot wounds resulting in hospitalization in the United States: 2004-2013. Injury. 2017;48(3):621-627. doi:10.1016/j.injury.2017.01.044 28173921

[16] Gani F, Sakran JV, Canner JK. Emergency department visits for firearm-related injuries in the United States, 2006-14. Health Aff (Millwood). 2017;36(10):1729-1738. doi:10.1377/hlthaff.2017.0625 28971917

[17] Kreimeyer K, Foster M, Pandey A, . Natural language processing systems for capturing and standardizing unstructured clinical information: a systematic review. J Biomed Inform. 2017;73:14-29. doi:10.1016/j.jbi.2017.07.012 28729030 PMC6864736

[18] Zhou L, Baughman AW, Lei VJ, . Identifying patients with depression using free-text clinical documents. Stud Health Technol Inform. 2015;216:629-633. 26262127

[19] Blackley SV, MacPhaul E, Martin B, Song W, Suzuki J, Zhou L. Using natural language processing and machine learning to identify hospitalized patients with opioid use disorder. AMIA Annu Symp Proc. 2021;2020:233-242. 33936395 PMC8075424

[20] Pedregosa F, Varoquaux G, Gramfort A, . Scikit-learn: machine learning in Python. J Mach Learn Res. 2011;12:2825-2830.

[21] Parker ST. Estimating nonfatal gunshot injury locations with natural language processing and machine learning models. JAMA Netw Open. 2020;3(10):e2020664. doi:10.1001/jamanetworkopen.2020.20664 33052403 PMC7557517

