# Supplementary material for: Classifying Firearm Injury Intent in Electronic Hospital Records Using Natural Language Processing
Source: JAMA Netw Open. 2023 Apr 6;6(4):e235870. doi: 10.1001/jamanetworkopen.2023.5870 (PMC10080369; doi:10.1001/jamanetworkopen.2023.5870)
Supplement: Supplement 1. — eAppendix 1. CODE BOOK (October 5, 2022) eAppendix 2. ICD Codes Used to Identify Gunshot Injuries eTable 1. NLP Lexicon Categories and Sample Terms eTable 2. Rule System Descriptions eTable 3. Comparison of NLP Models on Tune Set eTable 4. Top (and Bottom) 5 Highest (and Lowest) Weighted Rules [file jamanetwopen-e235870-s001.pdf]

Supplementary Online Content

MacPhaul E, Zhou L, Mooney SJ, et al. Classifying firearm injury intent in electronic hospital records using natural language processing. *JAMA Netw Open*. 2023;6(4):e235870. doi:10.1001/jamanetworkopen.2023.5870

eAppendix 1. CODE BOOK (October 5, 2022)

eAppendix 2. *ICD* Codes Used to Identify Gunshot Injuries

eTable 1. NLP Lexicon Categories and Sample Terms

eTable 2. Rule System Descriptions

eTable 3. Comparison of NLP Models on Tune Set

eTable 4. Top (and Bottom) 5 Highest (and Lowest) Weighted Rules

This supplementary material has been provided by the authors to give readers additional information about their work.

© 2023 MacPhaul E et al. *JAMA Network Open.*

eAppendix 1. CODE BOOK (October 5, 2022)

Legend: Variable names are noted in **ALLCAPS**. Words that are in all caps but are not bold and have an asterisk in them (e.g., *PHRASE), indicate that a repeating variable or parallel variables will appear directly below.

1. **Case Definition Variables**: Review the relevant portions of the electronic health record and code three variables to establish whether the case meets the project **case definition** as an initial encounter for an injury resulting from a projectile fired from a firearm:
   a. **GSW** *Definition*: Injury resulted from a traditional firearm projectile (non-traditional projectile defined below) fired from a firearm
      1 Yes
      0 No/unknown

   b. **NOTGSW** (if GSW=0) *Definition*: Type of injury or condition if not a firearm projectile injury
      1 Not a firearm (e.g., BB gun, air rifle, nail gun, paint gun, Verey pistol, flare gun, pellet gun etc.)
      2 Non-projectile firearm injury (e.g., pistol whipping, injury to thumb webbing from pistol slide, burn from spent casing)
      3 Non-traditional projectile (e.g., beanbag ammo, rubber bullet)
      4 Other (medical condition, other type of injury)
      9 Unknown

   c. ARRIVAL *Definition*: How did the patient arrive at the ED?
      1. Ambulance or Airlift (EMS)
      2. Self referral, private vehicle, etc.
      3. Law Enforcement
      4. Transfer from Another Hospital
      5. Other

   d. **FIRST** *Definition:* Was this the first episode of care? The objective of the project is to determine the number of hospital-treated gunshot injuries that occur by intent. To that end, to avoid the double-counting that would result if multiple visits for the same injury were treated as cases, we code only those types of presentation listed below as first encounters .
      1 Yes (first episode of care following injury; includes ED presentation, transfer from another ED, admission from ED. If someone has never sought care and is now presenting for wound infection, that counts as first episode)
      0 No (e.g., subsequent care after first ED visit/transfer/admission, e.g., for suture removal, wound check, wound infection, sequelae of injury, late effects. If a person visited a hospital earlier, walked out and visited another hospital for the same injury, the second visit=0 for first episode of care)
      9 Unknown

Continue with remaining steps only if GSW=1 and FIRST=1

© 2023 MacPhaul E et al. *JAMA Network Open.*

2. **Intent Classification**: (If GSW=1 and FIRST=1) Enter five variables to classify intent and indicate the specific phrases that led to that classification.

   a. INTENT *Definition*: Classification of firearm injury intent.
   See detailed definition of each intent in Coding Guidance, p. 6.

1	SUICIDE/INTENTIONAL SELF-HARM
2	ASSAULT/INTERPERSONAL VIOLENCE
3	ACCIDENT/UNINTENTIONAL
4	LEGAL INTERVENTION
8	UNABLE TO DETERMINE DUE TO CREDIBLE CONFLICTING EVIDENCE
9	UNKNOWN – NOT ENOUGH INFORMATION

b. ***PHRASE** Five free-text fields into which abstractor types verbatim the key, specific phrases from the EHR that provide information about intent. Include all statements relevant to intent even if they do not support the intent type ultimately coded. For each of the free text fields, indicate the location of the intent information, as described below. Some examples of phrases:

- "Shot on street while walking with friend; friend also shot and died."
- "Shot by unknown assailant."

IntentLocation1

First Location of intent information in EHR:

1 Outside Report: records from sources such as ambulance, airlifts
2 ED Notes
3 Inpatient Notes
4 Discharge Summary
5 Other

TextField1

Please copy the relevant phrases from the above location into the box.

IntentLocation2

Second Location of intent information in EHR:

1 Outside Report: records from sources such as ambulance, airlifts
2 ED Notes
3 Inpatient Notes
4 Discharge Summary
5 Other

TextField2

Please copy the relevant phrases from the above location into the box.

IntentLocation3

Third Location of intent information in EHR:

1 Outside Report: records from sources such as ambulance, airlifts
2 ED Notes
3 Inpatient Notes
4 Discharge Summary

© 2023 MacPhaul E et al. *JAMA Network Open.*

5 Other

TextField3
Please copy the relevant phrases from the above location into the box.

IntentLocation4
Fourth Location of intent information in EHR:
1 Outside Report: records from sources such as ambulance, airlifts
2 ED Notes
3 Inpatient Notes
4 Discharge Summary
5 Other

TextField4
Please copy the relevant phrases from the above location into the box.

IntentLocation5
Fifth Location of intent information in EHR:
1 Outside Report: records from sources such as ambulance, airlifts
2 ED Notes
3 Inpatient Notes
4 Discharge Summary
5 Other

TextField5
Please copy the relevant phrases from the above location into the box.

d. AXIS* (only if INTENT=8) *Definition:* Which intent types conflict? For example, if the injury was clearly self-inflicted but unclear whether intentional or unintentional, code AXIS1="1, suicide/intentional self-harm" and AXIS2="3, accident."
AXIS1 (use INTENT code list)
AXIS2 (use INTENT code list, or choose "7" if there is more than one alternate intent being considered beyond AXIS1)

4. **Circumstance leading to shooting**: Code the circumstance of the incident in one of four intent-specific *CIRC Intent-specific circumstance associated with the shooting. *Code just one variable below, or code none if INTENT= 8 or 9 (unknown).*

SUICCIRC (if INTENT=1) *Definition*: Type of intentional self-harm.
1 Suicide attempt
2 Non-suicidal self-harm
9 Unknown

VIOLCIRC (if INTENT=2) *Definition*: Precipitating circumstance of the Assault/Interpersonal Violence.
1 Intimate partner or family violence
2 Felony-type crime (occurred during the commission of a, or in reference to, a non-IPV/DV felony, like robbery, drug trade, car-jacking)

© 2023 MacPhaul E et al. *JAMA Network Open.*

3 Gang-related (other than IPV/DV or felony-type)
4 Argument (e.g., drunk people arguing at party, road rage, revenge over sleight)
5 Law enforcement officer injured on active duty
7 Other precipitating circumstance (e.g., psychotic shooting, justifiable)
9 Unknown

ACCIDCIRC (if INTENT=3) *Definition:* Context of Accident/Unintentional shooting.

1 Playing/fooling around with gun
2 Hunting or target shooting accident
3 Cleaning gun
4 Holstering/unholstering gun (includes removing/returning to holster,pocket, waistband)
5 Other functional handling (e.g. while transporting, showing gun)
7 Other unintentional discharge
9 Unknown

LEGALCIRC (if INTENT=4) *Definition*: Type of circumstance that brought police and pt. together.

1 Mental health call (e.g., suicide threat, naked guy yelling in street)
2 Intimate partner/family violence or disturbance (other than mental health call)
3 Felony-type crime
4 Traffic stop
7 Other
9 Unknown

5. **Relationship of Shooter to Patient**

a. **SELF** *Definition*: Shot by self, someone else, or unknown

0 Someone else
1 Self
9 Unknown

b. **RELATION** *Definition*: Relationship of shooter to patient, if shot by another person (e.g., patient was shot by x)

1 Current or former intimate partner
2 Other family
3 Acquaintance (neighbor, classmate, friend of a friend)
4 Police
5 Stranger
6 Unspecified (unknown)
7 Known shooter

6. ADDITIONAL CONTEXT CODES:

a. **MULTIVICT** *Definition*: more than one person was shot during the incident

0 No
1 Yes

b. **MULTISHOTS** *Definition:* Multiple shots (either multiple bullet wounds or heard multiple shots). If multiple GSWs from one bullet (e.g. single bullet causing an entry and exit wound), code as no; if multiple gunshot wounds and it is unknown whether from a single or multiple shots, code as yes.

0 No or unknown
1 Yes

c. **WITHHOLD** *Definition:* Patient appears to be withholding information (e.g., pt refuses to speak; pt uses some version of "I was walking along, heard some pops, and next thing I knew…")
   0 No or unknown
   1 Yes

d. **2STORIES** *Definitio*n: Conflicting stories or evidence point to conflicting intent types (regardless of the credibility of that evidence).
   0 No or unknown
   1 Yes
e. **DRIVEBY** *Definition:* Shooter was riding in/on a vehicle (car, bicycle, truck) when he/she shot pt and patient was not in the car with the shooter.
   0 No or unknown or not applicable (for example, if the patient shot themselves)
   1 Yes
f. **PLACE** *Definition*: Where pt. was when the shooting occurred.
   1 House, apartment, including driveway, porch, yard, garage
   2 Street, sidewalk
   3 Motor vehicle (victim was sitting in, driving, getting in or out of MV, regardless of where vehicle is located)
   4 Parking lot/public parking garage
   5 Public transportation or station (e.g., bus, train, plane, airport, depot, taxi)
   6 Bar, nightclub
   7 Service station
   8 Liquor store
   9 Hotel/motel
   10 Other commercial establishment
   11 Public institution (e.g., hospital, prison, house of worship, social service agency, etc.)
   12 Sports or athletic area (e.g., stadium, baseball field, gymnasium, recreation center)
   13 Park, playground, public use area
   14 Farm
   15 Natural area (e.g., field, river, beach, woods)
   16 Shooting area (e.g., shooting range, sportsman club)
   17 Party, unspecified location
   77 Other
   99 Unknown
g. **ACTIVITY** *Definition*: What activity was the patient doing when they were shot? (These are ICD activity codes)
   1 While engaged in sports activity
   2 While engaged in leisure activity
   3 While working for Income
   4 While engaged in other types of work (e.g., chores, school)
   5 While resting, sleeping, eating, or engaging in other vital activities
   6 While involved in altercation or fight
   7 While engaged in other specified activities
   8 While engaged in unspecified activities

© 2023 MacPhaul E et al. *JAMA Network Open.*

h. **Location of Injury** *Definition*: Where are the GSW(s) on the body? (each of these are separate 0/1 variables)

HEAD
FACE
NECK
UPPEREXT – hand, arm, shoulder (LUE, RUE)
BACK- spine
CHEST - thorax
ABDOMEN- flank
LOWEREXT – leg, foot, buttocks

i. **DISPOSITION** *Definition*: Describe the final outcome of the hospital stay We code for, transfers, admissions and deaths so that they can be included or omitted as necessary in counts (e.g., if one were counting the number of gunshot injuries that were treated at hospitals, it would be appropriate to include injuries that resulted in death; this would not be the case if the objective were to count non-fatal injuries only).

1 Discharged from ED (to home, police custody, etc.)
2 Admitted to hospital associated with ED and discharged from hospital
3 Died
4 Transferred to another acute care hospital
7 Other
9 Unspecified

j. **LOCDEATH** (If Outcome = 3, Died) Where patient died.

1 Dead on arrival
2 Emergency department
3 Inpatient unit
4 Other/unspecified

© 2023 MacPhaul E et al. *JAMA Network Open.*

CODING GUIDANCE (INTENT)

Intentional Self-harm (1)

Any self-inflicted injury resulting from the purposeful use of force against oneself. This category includes both suicide attempts and non-suicidal intentional self-harm. The variable SELFCIRC allows the coder to distinguish between suicide attempts and non-suicidal self-harm, when known.)

Use of the phrase "self-inflicted" alone can generally be interpreted to mean "intentionally self-inflicted" if the record does not indicate it was an accident.

The fact that an intentional, self-inflicted act was undertaken while under the influence of a mental illness or mind-altering drug does not change the classification.
Russian roulette is classified as Suicide/Intentional Self-harm.
"[Attempted] suicide by cop" should be classified as Legal Intervention, not Suicide/Intentional Self-harm, if the shot was taken by police.
If a person shoots his or herself while fleeing from, or engaged in a conflict with, police, classify the act as Suicide/Intentional Self-harm, not Legal Intervention.

Examples of SUICIDE/INTENTIONAL SELF-HARM (followed by how SUICCIRC should be coded for that case):

- "Pt attempted suicide with shotgun blast to head." *SUICCIRC=1 (Suicide Attempt)*
- "GSW to head after long struggle with bipolar disorder." *SUICCIRC=1*
- "Pt shot self in front of his ex-girlfriend after attempted reconciliation." *SUICCIRC=1*
- "Pt shot himself in the heart; was due to be sentenced the next day and said he would not return to prison." *SUICCIRC=1*
- "Police were engaged in motor vehicle chase after pt robbed a convenience store. Pt pulled over and shot self in the head." *SUICCIRC=1*
- "Pt had been drinking at a party and was playing Russian roulette." SUICCIRC=1
- "Pt under the influence of meth shot knee because he was angry about pain in his knee." *SUICCIRC=2 (Non-suicidal self-harm)*
- "Pt with history of schizophrenia shot herself in abdomen in attempt to abort pregnancy." (SUICCIRC=2
- "Self-inflicted gunshot wound" (provided there is no evidence that it was accidental)
- Gun found on scene, suicide note found
- "impulsive" act of self-inflicted gsw in setting of frustration from dispute

Assault/interpersonal violence (2)

For this project, we will default to assigning all cases to assault/interpersonal violence if the case does not meet the criteria for intentional self-injury, unintentional injury, legal intervention, or injury of unknown intent, per the guidance provided here.

Cases with more than one victim (VICTIMS=1) or multiple gun shots (MULTIPLE=1) should be coded as assault, even if no additional information is available.

© 2023 MacPhaul E et al. *JAMA Network Open.*

Code shootings with information about any of the following as assault: shot by (person x), shot while (activity x), or shot at (location x).

Code as assault cases that don't have any circumstantial information, but mention the terms: assault, assailant, perpetrator, victim, violence, or referrals to violence recovery program

If no circumstantial information about the shooting is present, but the police are treating the incident as a homicide, looking for suspect, or if someone was arrested for the incident, code as assault.

NB: for cases in which there is sufficient circumstantial information about the shooting incident provided to determine, in order to code assault:

1) the gunshot wound itself need not have been intentionally inflicted to be classified as assault, but the use of the firearm to threaten, harm, or defend against another person must be. For example, say someone shoots up a building to threaten its residents, but shots penetrate the wall and a resident is unintentionally injured by a bullet. The case will be classified as assault even though the injury was unintended.
2) Gunshot injuries inflicted by a law enforcement officer are separately classified (as below).

Examples of cases to be coded as assault/interpersonal violence include:

- "Pt shot by clerk when he was attempting to rob a liquor store"
- "Bystander was unintentionally shot during gunfight between two others"
- "Pt was shot during a drug deal"
- "Pt was a police officer who was shot while attempting to serve a search warrant"
- "Police are treating the case as an attempted murder"
- "Pt. states was walking home when someone started shooting at him; cannot identify person"
- "Involved in altercation, sustained GSW"
- "Victim of community violence" "Victim of gun violence"
- Mention of assault, assailant (e.g., "shot by unknown assailant")
- "The police suspect the shooting was gang-related"
- "Pt reports not having a history with perpetrator"
- "Leaving a party when unknown person began shooting; doesn't believe she was the intended target"
- "Pt states he was walking along and heard a shot and realized he was hit"
- "Pt was getting out of his car when he was shot"
- "Pt was shot at while she was standing with friends at the park"
- "Shot from a passing car"
- "GSW to left shoulder; pt states he wasn't the intended target"
- No information about incident, but referrals or discharge planning implies violence (e.g., "Pt referred to violence interruption program," "Pt believes it will be safe to return to his neighborhood"
- "Pt reports he was drinking alcohol and he only heard one shot."
- Pt was standing and was shot in the RLQ with a 22 caliber gun.

© 2023 MacPhaul E et al. *JAMA Network Open.*

Accident/unintentional (4)

Injury resulted from the unintentional discharge of a firearm (e.g., shooter mistakenly pulled trigger) or from the intentional (but not violence-related) discharge of a gun if the shooter was unaware that an injury would occur (e.g., pt shot when a fellow hunter was swinging on prey; teenager shot when fooling around with a gun he thought was unloaded). Accidents can be self-inflicted or inflicted by another person. If a patient unintentionally shot self when using a gun to defend against or threaten another person, code that as an accident. If someone else was injured, code it as assault/interpersonal violence).

Examples:

- Celebratory firing that was not intended to frighten, control, or harm anyone (e.g., "Neighbors were shooting in celebration on 4th of July; pt was standing on a balcony and was hit.")
- Child under age 6 shoots self or another person (too young to be considered intentional)
- "Pt. stumbled and shot foot while in yard with gun drawn to investigate suspicious noises"
- "Two youths were playing 'quick draw' with pistols they thought were unloaded."

Indistinct language – Sometimes, given the context, the intent is implied but not explicitly stated. When a shooting was stated as occurring while the shooter was cleaning or repairing a gun, target shooting, engaged in hunting or pest control, playing with a gun (other than in a Russian roulette type game or a threatening game), or during routine handling, intent can be noted as "accident" if no evidence exists to the contrary.

Examples:

- "Pt shot during a hunting incident"
- "Pt sustained GSW while pulling rifle from truck bed of her pickup"
- "GSW to left shoulder while in woods behind outdoor shooting range"
- "GSW while dismounting a tree blind"
- "Pt was injured while cleaning his handgun"
- "Pt. and friends were horsing around with a gun"
- "patient's friend was showing his gun to the patient and it discharged"
- "gun misfired while patient's sister was trying to remove the gun from patient to prevent him from harming himself"

Legal Intervention (4)

Code shootings as legal intervention if the shooter was a sworn officer who was acting in the line of duty. A "*sworn officer*" includes someone employed by a public agency with statutory authority to use lethal force, including a police officer, sheriff, sheriff's deputy, corrections officer, federal law enforcement officer (e.g. FBI, secret service, drug enforcement agent, ATF agent), military police, animal control officer. It does not include a private security guard. An incident occurred "*in the line of duty"* if the officer was on duty (provided the case was not a clear murder, such as an on-duty officer who murders his or her spouse) or if an off-duty officer interceded as an officer. Some shootings in the line of duty may seem unjustifiable; coding them as legal interventions does not imply that they were necessarily justifiable, simply that they were related to legal intervention. Finally, in some cases an off-duty officer is personally the victim of a crime and responds by shooting the alleged offender. These cases will be coded as assault/violence-related, not legal intervention (e.g., off duty officer encounters a person who is trying to break into officer's home and shoots him).

© 2023 MacPhaul E et al. *JAMA Network Open.*

In unusual circumstances, some “legal intervention” cases will be accidental, not interpersonal violence-related, but these should still be coded as “legal intervention” (e.g., pt was shot when a police officer shot at a bear).

Examples:

- Pt. shot by police during an attempted carjacking.
- Pt had someone at gunpoint in a bar. Off-duty officer who was working security announced himself as an officer and shot pt when pt refused to drop the gun.
- Pt was a police officer who was hit by “friendly fire” from another officer during a foot chase with a suspect.
- Pt was shot while attempting to escape from prison.
- Pt was shot during a routine traffic stop when police thought he was reaching for a gun.

Unable to determine due to conflicting evidence (8)

Resolvable conflicting cases:
When there is suggestive evidence for more than one intent type and abstractor **can find some basis** for deciding between them.
Examples:

- “Pt. on arrival states this was an assault and later stated he’d actually shot himself accidentally and had been too embarrassed to admit it”
- “Patient sustained GSW to head while in garage with brother. Unknown is self-inflicted. Police say patient was “accidentally” shot by pt’s friend, but case is being investigated as a homicide”

Unresolvable conflicting cases:
When there is suggestive evidence for more than one intent type and abstractor **can find no basis** for deciding between them.

Examples:

- “Notes from social worker indicate she thinks this may be an accident; notes from EMS state woman shot in DV incident, police arrested husband.”

Unknown (not enough information) (9)

Code shootings as unknown (9) when nothing is known about the circumstances of the shooting and no information in the record suggests that the injury was the result of an assault. The fact that police were involved does not necessarily mean the case was an assault.

Example:

- “Pt with GSW to the head transferred from Mercy Hospital; circumstances unknown.”
- “Pt found in ditch, GSW to chest”
- “Found down in street”
- “Pt who is under house arrest transferred by EMS after GSW to foot under unclear circumstances.“

© 2023 MacPhaul E et al. *JAMA Network Open.*

- “Pt and friend report to the ED; friend was reportedly without injuries, but patient had gsw to head.”
- “Pt is not sure exactly when the gun shot happened, he does not know who shot him. He states at the time of the shot he "blacked-out". He states he was at home and noticed his left thigh hurt and then he was at a hospital.”
- “Pt admitted with GSW to back. Circumstances of shooting remain unclear. Police are involved and interviewed pt in ED.”
- “Pt arrives with a GSW to left arm. Pt declined to answer questions. History limited by patient’s unwillingness to participate.”
- “Pt states he was shot in the abdomen”
- “Pt presents with GSW to the chest. Police are involved.

© 2023 MacPhaul E et al. *JAMA Network Open.*

eAppendix 2. *ICD* Codes Used To Identify Gunshot Injuries

We identified all patients who presented to an emergency department for whom the clinical record included an ICD code indicating the patient had suffered a firearm injury in any diagnosis field: codes E965 (0.0-0.4, 0.9), E979.4, E955(0.0-0.9), E922 (0.0-0.3, 0.8, 0.9), E985 (0.0-0.4), E970, for all records falling under ICD-9-CM, i.e., prior to 10/1/15; and for all subsequent periods, ICD-10-CM codes: W320XX-WW321XX, W330XX-W3309X, W3309X-W3313X, W3319X, W3400X, W3409X-W3410X, W3419X, X72XXX, X730XX-X732XX, X738XX-X739XX, X748XX-X749XX, Y384X1-Y384X3, X93XXX, X940XX-X942XX, X948XX-X949XX, X958XX-X959XX, Y22XXX, Y230XX-Y233XX, Y238XX-Y239XX, Y248XX-Y249XX, Y35001-Y35003, Y35009, Y35011-Y35013, Y35019, Y35021-Y35023, Y35029,Y35031-Y35033, Y35039, Y35091-Y35093, and Y35099.

Lexicon

term	term_group	info1	info2
accident	accident		
accidental	accident		
accidental self inflicted	accident		
accidental selfinflicted	accident		
accidentally	accident		
accidentally	accident		
accidentally self inflicted	accident		
accidentally selfinflicted	accident		
friendly fire	accident		
inadvertent	accident		
inadvertently	accident		
incident	accident		
incidentally	accident		
unintentional	accident		
unintentionally	accident		
assault	assault		
assaulted	assault		
community violence	assault		
drive by	assault		
gunfight	assault		
homicide detective	assault		
referred to violence interruption program	assault		
retaliation	assault		
targeted	assault		
viap	assault		
victim of violence	assault		
violence intervention advocacy program	assault		
violence recovery	assault		

© 2023 MacPhaul E et al. *JAMA Network Open.*

violence recovery program	assault	
vov	assault	
vrp	assault	
wrong place at the wrong time	assault	
arm	body	arm
finger	body	arm
hand	body	arm
upper extremity	body	arm
back	body	back
shoulder	body	back
spine	body	back
abdomen	body	chest
chest	body	chest
heart	body	chest
lung	body	chest
torso	body	chest
brain	body	head
chin	body	head
face	body	head
head	body	head
neck	body	head
buttock	body	leg
calf	body	leg
foot	body	leg
hip	body	leg
knee	body	leg
leg	body	leg
lower extremity	body	leg
thigh	body	leg
robbed	crime	assault
stabbed	crime	assault
carjacking	crime	assault
crime	crime	assault
drug deal	crime	assault
robbery	crime	assault
cleaning	event	accident
holster	event	accident
hunting	event	accident
misfired	event	accident
putting away	event	accident

© 2023 MacPhaul E et al. *JAMA Network Open.*

ricocheted	event	accident
taking apart	event	accident
unjam	event	accident
altercation	event	assault
confrontation	event	assault
fight	event	assault
gunpoint	event	assault
walking	event	assault
argument	event	assault; suicide
break up	event	suicide
breaking up	event	suicide
divorce	event	suicide
drinking	event	suicide
found	event	suicide; undetermined
bullet	gun	
firearm	gun	
gun	gun	
handgun	gun	
pistol	gun	
rifle	gun	
sgw	gun	
shotgun	gun	
weapon	gun	
went off	gunshot	accident
discharged	gunshot	accident
opened fire	gunshot	assault
a pop	gunshot	
bang	gunshot	
fired	gunshot	
firing	gunshot	
gsw	gunshot	
gunfire	gunshot	
gunshot	gunshot	
pops	gunshot	
shooting	gunshot	
shot	gunshot	
left hand dominant	handedness	accident
left handed	handedness	accident
lhd	handedness	accident
rhd	handedness	accident

© 2023 MacPhaul E et al. *JAMA Network Open.*

right hand dominant	handedness	accident	
right handed	handedness	accident	
sustained	injury	accident	
injured	injury		
injury	injury		
puncture	injury		
wound	injury		
wounded	injury		
beanbag	legal		
rubber bullet	legal		
apartment	location	inside	
home	location	inside	
house	location	inside	
parking lot	location	outide	
gun range	location	outside	accident
shooting range	location	outside	accident
car	location	outside	
ditch	location	outside	
park	location	outside	
porch	location	outside	
store	location	outside	
street	location	outside	
van	location	outside	
bipolar disorder	mental health		
depressed	mental health		
depression	mental health		
depressive	mental health		
grief	mental health		
intoxicated	mental health		
air rifle	notgun		
bb gun	notgun		
flare gun	notgun		
nail gun	notgun		
paintball gun	notgun		
pellet gun	notgun		
herself	person	self	
himself	person	self	
self	person	self	
themself	person	self	
assailant	person	shooter	assault

© 2023 MacPhaul E et al. *JAMA Network Open.*

gang	person	shooter	assault
perpetrator	person	shooter	assault
unknown assailant	person	shooter	assault
officer	person	shooter	legal
police	person	shooter	legal
police officer	person	shooter	legal
bystander	person	victim	assault
intended target	person	victim	assault
victim	person	victim	assault
friend	person		accident
attempted suicide	suicide		
ideation	suicide		
past attempt	suicide		
russian roulette	suicide		
self-inflicted	suicide		
self inflicted	suicide		
selfinflicted	suicide		
suicidal	suicide		
suicidal ideation	suicide		
suicide	suicide		
suicide attempt	suicide		
does not recall	undetermined		
reluctant	undetermined		
not clear	undetermined		
poor historian	undetermined		
unclear	undetermined		
police report	unhelpful		
unloaded	accident		
not loaded	accident		
exploded	gunshot	accident	
playing with	event	accident	
unknown pmh	undetermined		
unknown past medical history	undetermined		
unidentified	undetermined		
single gunshot wound	gunshot	undetermined	
single gsw	gunshot	undetermined	
unconscious	undetermined		
unclear	undetermined		

© 2023 MacPhaul E et al. *JAMA Network Open.*

eTable 1. NLP Lexicon Categories and Sample Terms

Term Category	Sample Terms
Accident	Accident, Accidentally, Incidentally, Unintentional
Assault	Assault, Assailant, Community Violence, Victim of Violence
Body	Arm, hand, back, shoulder, spine, head, neck, leg
Crime	Robbed, Stabbed, Carjacking, Drug Deal
Event	Hunting, Cleaning, Fight, Divorce, Drinking
Gun	Firearm, Pistol, Handgun, Weapon
Gunshot	Pops, bangs, shots, gunfire, discharged, single gsw, multiple shots
Handedness	Left-handed, right hand dominant, lhd
Injury	Injury, Wound, Punctured
Legal	Police, Beanbag, Rubber Bullet
Location	Apartment, Home, Parking lot, Car, Street
Mental Health	Depression, Bipolar Disorder, Grief, Intoxicated
Person	Self, perpetrator, bystander, intended target, friend
Self-Harm	Attempted suicide, suicidal ideation, self-inflicted
Undetermined	Unconscious, unclear, poor historian, reluctant
Shooting Verbs	Firing, Fired, gsw, gunshot, shooting, shot, sustained
Confusion Terms	Air rifle, bb gun, flare gun, paintball gun, police report

© 2023 MacPhaul E et al. *JAMA Network Open.*

eTable 2. Rule System Descriptions

Name	Description	Type
explicit_{all intents}*	True if the sentence contains a term from the lexicon in the explicit intent category, such as "accidentally" for the explicit accident rule	rule
implicit_{all intents}*	True if the sentence contains any term from the lexicon tagged with the intent, such as "cleaning a gun" and the implicit accident rule	rule
is_gsw	True if a non-negated gunshot injury term is found or if an injury term and a gun term are both found	rule
heard_shots	True if the word "heard" is not negated and in a gunshot term's context	rule
multiple_shots	True if a plural gunshot term is found or if multiple gsw lexicon terms are found	rule
if_circumstances	True if the find circumstances parser returned any information	rule
crime_circumstances	True if the text returned by the find circumstances parser contains any terms in the crime category	rule
accident_event	True if the shooting was preceded by an event category term with the accident tag, such as cleaning one's gun.	rule
suicide_event	True if the shooting was preceded by an event category term with the suicide tag	rule
found_outside	The patient was found down at a location tagged outside	rule
found_inside	The patient was found down at a location tagged inside	rule
outside	The shooting occurred at a location tagged outside	rule
inside	The shooting occurred at a location tagged inside	rule
in_car	Incident occurred while patient was in a car	rule
accident_place	Incident occurred at a place with the accident tag	rule
handedness	The narrative text references the patient's dominant hand while describing the shooting	rule

© 2023 MacPhaul E et al. *JAMA Network Open.*

mental_health	Mention of mental health issues	rule
self_victim	The text found by the find victim parser contains a term with the self tag	rule
assault_shooter	The shooter is described using lexicon terms with the assault tag, such as "assailant"	rule
body_head	The patient was shot in the head	rule
body_chest	The patient was shot in the chest	rule
body_back	The patient was shot in the back	rule
body_arms	The patient was shot in the arms or hands	rule
body_legs	The patient was shot in the legs or feet	rule
high_info_note	The case has at least one note with more lexicon terms than most notes (more than 20 terms in unique contexts)	rule
credible_conflicting	The case has multiple explicitly stated intents	rule
low_info_case	The case has fewer lexicon terms than most cases (fewer than 200 terms in unique contexts)	rule
not_explicit	True if no explicit intent rules returned true	rule
_find_shooter	Finds the shooter by checking the subject of shooting verbs	parser
_find_victim	Finds the victim by checking the object of shooting verbs	parser
_find_circumstances	Finds adverb clauses and preposition phrases tied to shooting verbs	parser

*one version of this rule exists separately for each intent

© 2023 MacPhaul E et al. *JAMA Network Open.*

eTable 3. Comparison of NLP Models on Tune Set

		Rfc	Maxent	Svm	knn	gbc	Ada
Ac	P	.6667	.4681	.5116	.5	.8148	.2706
	R	.6429	.7857	.7857	.5	.7857	.8214
	F	.6545	.5867	.6197	.5	.8	.4071
	ROC	.9292	.9623	.9608	.8347	.9612	.8834
	PRC	.6377	.6947	.7393	.5059	.73	.4561
As	P	.8612	.9502	.9296	.8191	.8780	.8848
	R	.8897	.7022	.7279	.8493	.9265	.6213
	F	.8752	.8076	.8165	.8339	.9016	.7300
	ROC	.8611	.8946	.8821	.8385	.8969	.7827
	PRC	.9310	.9486	.9387	.9020	.9473	.8791
Ll	P	1	.4667	.7	0	1	.1667
	R	.75	.875	.875	0	.875	.875
	F	.8571	.6087	.7778	0	.9333	.28
	ROC	.9166	.9388	.9178	.8669	.9520	.9247
	PRC	.8620	.8259	.8789	.4186	.8815	.8792
SH	P	.7917	.625	.7333	.7391	.8333	.6296
	R	.7917	.8333	.9167	.7083	.8333	.7083
	F	.7917	.7143	.8148	.7234	.8333	.6667
	ROC	.9920	.9723	.9863	.9451	.9849	.9484
	PRC	.8914	.8168	.7901	.7304	.8623	.6148
Un	P	.48	.4194	.4023	.4364	.5581	.2326
	R	.4286	.6964	.6607	.4286	.4286	.1786

© 2023 MacPhaul E et al. *JAMA Network Open.*

	F	.4528	.5235	.5	.4324	.4848	.2020
	ROC	.7916	.7990	.8059	.7897	.8105	.5971
	PRC	.4015	.4789	.4341	.3629	.4409	.1822
Avg	P	.7599	.5859	.6553	.4989	.8169	.4369
	R	.7006	.7785	.7932	.4972	.7698	.6409
	F	.7263	.6482	.7058	.4980	.7906	.4572
	ROC	.8981	.9135	.9016	.8550	.9211	.8272
	PRC	.7447	.7530	.7562	.5840	.7724	.6023

© 2023 MacPhaul E et al. *JAMA Network Open.*

eTable 4. Top (and bottom) 5 highest (and lowest) weighted rules

Rank	MDS model	EVS model
1	implicit_assault	Self_victim
2	explicit_suicide	Implicit_assault
3	accident_event	Body_head
4	self_victim	Explicit_legal
5	high_info_note	Accident_place
…		
32	not_explicit	Not_explicit
33	suicide_event	High_info_note
34	low_info_case	Found_outside
35	implicit_undetermined	Check_gsw
36	crime_circumstances	Low_info_case

© 2023 MacPhaul E et al. *JAMA Network Open.*
