# Supplementary material for: Classifying Firearm Injury Intent in Electronic Hospital Records Using Natural Language Processing
Source: JAMA Netw Open. 2023 Apr 6;6(4):e235870. doi: 10.1001/jamanetworkopen.2023.5870 (PMC10080369; doi:10.1001/jamanetworkopen.2023.5870)
Supplement: Supplement 2. — Data Sharing Statement [file jamanetwopen-e235870-s002.pdf]

Data Sharing Statement

MacPhaul. Classifying Firearm Injury Intent in Electronic Hospital Records Using Natural Language Processing. *JAMA Netw Open*. Published April 06, 2023.
doi:10.1001/jamanetworkopen.2023.5870

Data
Data available: No

Additional Information
Explanation for why data not available: HIPPA protection. We will share the algorithim for the MLP-ML model.
